# Effect of Serum and Oxygen Concentration on Gene Expression and Secretion of Paracrine Factors by Mesenchymal Stem Cells

**DOI:** 10.1155/2014/601063

**Published:** 2014-12-29

**Authors:** Patrick Page, Joshua DeJong, Alaina Bandstra, Robert A. Boomsma

**Affiliations:** ^1^Department of Biology, Trinity Christian College, 6601 W. College Drive, Palos Heights, IL 60463, USA; ^2^Illinois College of Optometry, 3241 S. Michigan Avenue, Chicago, IL 60616, USA

## Abstract

Mesenchymal stem cells (MSC) secrete paracrine factors that may exert a protective effect on the heart after coronary artery occlusion. This study was done to determine the effect of hypoxia and serum levels on the mRNA expression and secretion of paracrine factors. Mouse bone marrow MSC were cultured with 5% or 20% serum and in either normoxic (21% O_2_) or hypoxic (1% O_2_) conditions. Expression of mRNA for vascular endothelial growth factor (VEGF), monocyte chemotactic protein-1 (MCP-1), macrophage inflammatory protein-1*α* (MIP-1*α*), MIP-1*β*, and matrix metalloproteinase-2 (MMP-2) was determined by RT-qPCR. Secretion into the culture media was determined by ELISA. Hypoxia caused a reduction in gene expression for MCP-1 and an increase for VEGF (5% serum), MIP-1*α*, MIP-1*β*, and MMP-2. Serum reduction lowered gene expression for VEGF (normoxia), MCP-1 (hypoxia), MIP-1*α* (hypoxia), MIP-1*β* (hypoxia), and MMP-2 (hypoxia) and increased gene expression for MMP-2 (normoxia). The level of secretion of these factors into the media generally paralleled gene expression with some exceptions. These data demonstrate that serum and oxygen levels have a significant effect on the gene expression and secretion of paracrine factors by MSC which will affect how MSC interact *in vivo* during myocardial ischemia.

## 1. Introduction

Work done in numerous laboratories has demonstrated that stem cell administration is able to reduce the loss of function in the heart after myocardial infarction. We have demonstrated that intravenous injection of bone marrow mesenchymal stem cells (MSC) into mice one hour after coronary artery occlusion is able to attenuate the loss of function due to myocardial infarction [1]. These MSC secrete the paracrine factors vascular endothelial growth factor (VEGF), monocyte chemotactic protein-1 (MCP-1), macrophage inflammatory protein-1*α* (MIP-1*α*), and MIP-1*β* which may play a role in the protective effect of MSC [2, 3]. Understanding the way in which the synthesis and secretion of these factors are controlled is important to better utilize MSC in clinical situations.

The amount of oxygen present while MSC are cultured can have a significant effect on the behavior of MSC [4]. The atmospheric oxygen level routinely used for culture (21% O_2_; normoxia) is significantly elevated compared to the 12% oxygen found in arterial blood and the 1–7% found in a variety of tissues like skin, bone marrow, myocardium, brain, and spleen [4–7]. In addition, after permanent coronary artery occlusion in rats, myocardial oxygen partial pressure levels drop from 15 mmHg (2%) to less than 2 mmHg (<0.3%) which is maintained after left ventricular remodeling [8]. Hypoxia, or “*in situ* normoxia” [9], alters MSC gene expression [10], affects the secretion of paracrine factors [11–13], and influences the ability of MSC to differentiate [14–16].

Similarly, the amount of serum present in the culture media affects stem cell behavior. MSC are generally cultured in 10–20% serum which contains numerous factors that may not be present in the tissues where these cells reside. In addition, the ischemic conditions present after coronary artery occlusion include both hypoxia and serum deprivation [17, 18]. Reduction of serum levels to 2% promotes cardiomyocyte differentiation [19], reduces cell proliferation, and upregulates genes for maintaining stemness, angiogenic factors, and endothelial differentiation [20] in MSC. Serum deprivation alters the secretion of paracrine factors and the expression of stem cell and endothelial markers in MSC [21–23].

We have previously demonstrated that MSC secrete VEGF, MCP-1, MIP-1*α*, and MIP-1*β* which have significant biological effects on cell migration, apoptosis, and capillary formation [2]. In addition, MMP-2 secreted by MSC may promote directed cell migration [24]. Therefore, the purpose of the present study was to mimic the conditions present during myocardial ischemia by lowering oxygen and serum concentrations during culture in order to determine the effects of these conditions on the gene expression and secretion of VEGF, MCP-1, MIP-1*α*, MIP-1*β*, and MMP-2 by bone marrow MSC. To that end, we cultured MSC in 5% or 20% serum in either a normoxic (21% O_2_) or hypoxic (1% O_2_) environment.

## 2. Methods

### 2.1. Mesenchymal Stem Cell Culture

Murine mesenchymal stem cells (MSC) were isolated from bone marrow and cultured in Mesencult basal medium with 20% murine serum supplement (Stem Cell Technologies) as described previously [1]. MSC (passages 3–10) were cultured at 37°C in 6-well plates in 2 mL/well Mesencult with 5% or 20% murine serum supplement at a concentration of 0.30 × 10^6^ cells/well. Normoxic cells were cultured for 48 hours under normal atmospheric oxygen (21%) plus 5% CO_2_. Hypoxic cells were cultured for 24 hours under normal atmospheric oxygen and then for an additional 24 hours in a reduced oxygen atmosphere (1% O_2_, 5% CO_2_, 94% N_2_).

A cell proliferation assay using alamarBlue (Invitrogen; DAL1025) was performed in order to correct for differences in growth rates under the different culture conditions. MSC were cultured as described above. After 48 hours of culture, the wells were washed with PBS and then treated with Mesencult + 5% serum supplement containing 10% alamarBlue reagent. Cells were incubated for 4 hours at 37°C under normoxic conditions. Absorbance was measured at 570 and 600 nm and the amount of alamarBlue reduction (AR) was determined. AR is directly proportional to the number of cells present.

### 2.2. Quantification of Paracrine Factor Secretion Using ELISA

After 48 hours of culture, media were removed and centrifuged at 14,000 ×g for 10 min. The supernatant was removed, separated into single use aliquots, and stored at −20°C. Media supernatants were analyzed by ELISA using Quantikine kits (R&D Systems) for VEGF (MMV00), MCP-1 (MJE00), MIP-1*α* (MMA00), MIP-1*β* (MMB00), and matrix metalloproteinase-2 (MMP-2; DMP2F0) according to manufacturer's directions. In order to correct for differences in cell proliferation and thus the amount of secretion produced per cell, all values were normalized to those measured for cells cultured in 5% serum under hypoxic conditions using the normalization factors determined by the alamarBlue cell proliferation assay (see below).

### 2.3. mRNA Quantification Using RT-qPCR

Total RNA was isolated after 48 hours of culture using RNAqueous-4PCR kits (Ambion). RNA was treated with DNAse I and then quantified and assessed for quality by measuring absorbance at 260 and 280 nm. Reverse transcription was performed on 1 *μ*g RNA using an iScript cDNA synthesis kit (BioRad) with blended oligo (dT) and random primers. qPCR was done on 2 *μ*L RT DNA template with iQ SYBR Green Supermix (BioRad) using the BioRad MJ Mini Thermal Cycler with MiniOpticon with 300 *μ*M specific primers (Table 1). No-RT and water controls were included to ensure specificity. After an initial hot start at 95°C (3 minutes), 40 cycles of 95°C (30 seconds), 55°C (30 seconds), and 72°C (1 minute) were performed. Single band products of the appropriate molecular size were confirmed for the primers using 2% agarose electrophoresis. Relative expression was determined using CFX Manager (BioRad) by comparing data to the reference gene YWHAZ (tyrosine 3-monooxygenase/tryptophan 5-monooxygenase activation protein, zeta polypeptide); this was then normalized to the average expression level.

### 2.4. Statistical Analysis

Mean and standard error were calculated for each treatment group, and significance was determined using Student's *t*-test. The following biologically relevant comparisons were made: (1) normoxic cells grown in 5% and 20% serum, (2) hypoxic cells grown in 5% and 20% serum, (3) normoxic cells with hypoxic cells grown in 5% serum, and (4) hypoxic cells with normoxic cells grown in 20% serum.

## 3. Results

### 3.1. Cell Proliferation Assay

Before the amount of paracrine factor secretion could be compared between treatment groups, differences in the number of cells present after 48 hours of culture had to be equalized. To do this, a cell proliferation assay using alamarBlue was performed. It was found that cells cultured with 20% serum supplement had significantly elevated levels of alamarBlue reduction (AR) (*P* < 0.01; *n* = 5) compared to 5% serum. While hypoxia had no effect on cells cultured in 5% serum, mean AR values were elevated in hypoxic conditions in 20% serum (*P* < 0.05; *n* = 5). Compared to cultures in 5% serum under normoxic conditions, the AR value was 24% higher in 20% serum/normoxic cultures and 36% higher in 20% serum/hypoxic cultures. Based on this data, ELISA values for 20% serum/normoxic cultures and 20% serum/hypoxic cultures were normalized to 5% serum/normoxic cultures by multiplying by 0.804 and 0.737, respectively. No correction factor was used for 5% serum/hypoxic cultures since AR values were not significantly different from 5% serum/normoxic cultures.

### 3.2. Paracrine Factor Secretion

**Figure 1:**
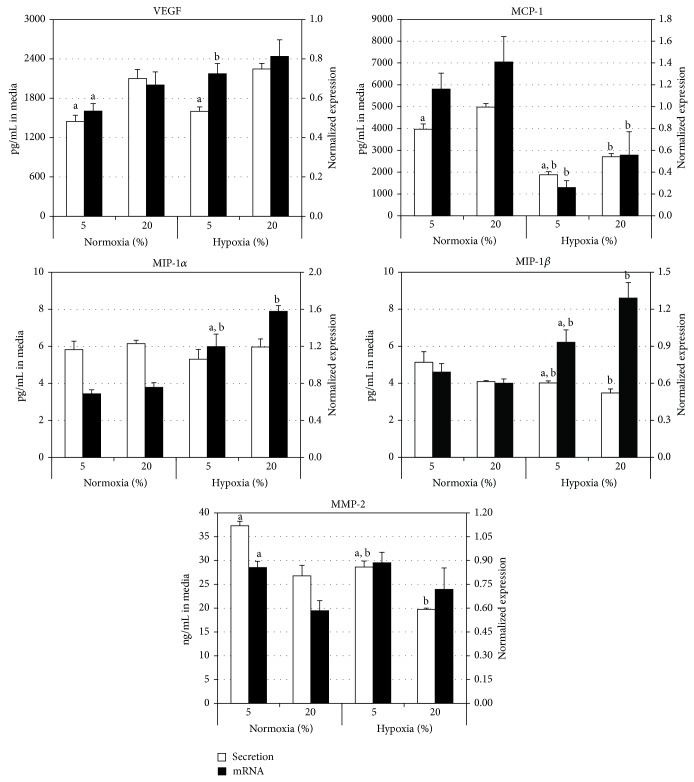
Normalized relative expression and secretion of paracrine factors under different oxygen and serum conditions. Data reported as mean ± SE. 5% and 20% refer to the amount of serum supplement present in the media. Open bars: secretion of factors into media. Solid bars: relative mRNA expression. (a) *P* < 0.05 versus 20%, (b) *P* < 0.05 versus normoxia.

The secretion of paracrine factors by MSC into the culture media was quantified using ELISA (Table 2, Figure 1). Serum reduction from 20% to 5% and changing from a normoxic to a hypoxic environment had significant effects on levels detected in the media. Previous studies in our laboratory have demonstrated that VEGF, MIP-1*α*, MIP-1*β*, and MCP-1 were not detectable in Mesencult media containing 20% serum supplement [2].

VEGF levels were significantly reduced (*P* < 0.01) in media from cells cultured in 5% serum supplement compared to those in 20% supplement in both normoxic and hypoxic conditions. Hypoxia had no effect on the secretion of VEGF.

As seen with VEGF, serum reduction to 5% significantly reduced (*P* < 0.01) MCP-1 levels in media compared to those in 20% supplement in both normoxic and hypoxic conditions. In addition, hypoxia significantly reduced (*P* < 0.01) MCP-1 levels in both 5% and 20% serum cultures.

MIP-1*α* levels were unaffected by serum supplement concentration or oxygen levels. On the other hand, MIP-1*β* levels (Table 2, Figure 1) were significantly elevated (*P* < 0.05) in 5% serum compared to 20% in hypoxic conditions, an effect not seen in normoxic conditions. Also, hypoxia significantly reduced (*P* < 0.05) MIP-1*β* secretion in both 5% and 20% serum supplements.

Changes in MMP-2 secretion followed the general trends seen for MIP-1*β*. Serum reduction to 5% resulted in a significant elevation (*P* < 0.01) of MMP-2 levels compared to 20% in both normoxic and hypoxic conditions. Also, hypoxia significantly reduced (*P* < 0.01) MMP-2 secretion in both 5% and 20% serum cultures.

### 3.3. mRNA Levels for Paracrine Factors

The normalized relative expression of mRNA in MSC for the various paracrine factors was determined using RT-qPCR (Table 3, Figure 1). Changes in mRNA paralleled changes in secretion although some differences were observed.

Serum reduction to 5% caused a significant drop in the relative expression of VEGF mRNA compared to 20% in normoxic (*P* < 0.05) but not in hypoxic conditions. Hypoxia resulted in a significant elevation of VEGF expression levels for cells grown in 5% serum (*P* < 0.05%) but not in 20%. This is similar to the response observed with VEGF secretion into the surrounding media except for the expression levels in 5% serum.

Serum reduction to 5% had no significant effect on the relative expression of MCP-1 mRNA compared to 20% serum supplement. Hypoxia significantly reduced (*P* < 0.01) the relative expression of MCP-1 mRNA in both 5% and 20% serum supplement. This was similar to what was observed for MCP-1 secretion except for the lack of an effect of serum.

Changes in the relative expression of MIP-1*α* and MIP-1*β* mRNA were similar to each other. Expression was significantly reduced (*P* < 0.05) in 5% serum compared to 20% in hypoxic conditions. No effect was seen in normoxic conditions. Hypoxia significantly elevated mRNA expression in both 5% (*P* < 0.05) and 20% (<0.01%) supplements. These effects were in contrast to secretion of these two factors into the culture media where hypoxia had no effect on MIP-1*α* and suppressed MIP-1*β* secretion.

Reducing the serum from 20% to 5% significantly increased (*P* < 0.05) the relative expression of MMP-2 mRNA in normoxic conditions similar to secretion into the media. However, reducing serum had no significant effect in hypoxia in contrast to secretion which showed an increase. Hypoxia had no effect on expression in either serum concentration while it reduced secretion into the media.

## 4. Discussion

This study clearly demonstrates that alteration of oxygen concentrations and serum levels results in significant changes in the mRNA expression for and secretion of paracrine factors by MSC. It is the first time that these two parameters have been studied simultaneously and the data suggests that the low serum and oxygen conditions present during the ischemia found after coronary artery occlusion may have significant effects on the secretory function of MSC and the role they play in preventing the loss of myocardial function after heart attack [1]. The particular changes observed depend on the specific paracrine factor being studied. Changes in mRNA often paralleled changes in secretion, but there were exceptions.

We found that hypoxia significantly reduced the gene expression for MCP-1 but significantly increased the expression of genes for MIP-1*α*, MIP-1*β*, and VEGF (5% serum only) while MMP-2 expression was unaffected. Secretions of MCP-1, MIP-1*β*, and MMP-2 were all significantly reduced by hypoxia while VEGF and MIP-1*α* were unaffected. The reasons for the differences between mRNA expression and secretion for MIP-1*α*, MIP-1*β*, and MMP-2 are not immediately apparent but may be due to differences in the control of gene expression versus control of translational and posttranslational events that lead from mRNA production to secretion. Other investigators have demonstrated an increase in VEGF gene expression and secretion [10–12] and a decrease in the secretion of MCP-1 [13] by MSC in hypoxia. Our results generally did not demonstrate an increase in VEGF after hypoxia, but this factor also responded differently to serum reduction in our hands (see below). In addition, hypoxia increased gene expression for placental growth factor, heparin-binding epidermal growth factor, MMP-9, and basic fibroblast growth factor (bFGF) [10, 12], caused an increase in the secretion of transforming growth factor-*β*2, insulin-like growth factor binding proteins 2, 3, 4, and 6, insulin-like growth factor- (IGF-) II, and interleukin-7 [11, 15], and reduced the secretion of stromal cell derived factor-1, macrophage colony stimulating factor, interleukin-1ra, RANTES, CXCL1, and CXCL10 [13] by MSC. Clearly, hypoxia changes the paracrine secretions of MSC which would have implications for the role they play in maintaining myocardial function after coronary artery occlusion.

Exposure to a hypoxic environment results in significant changes in cellular physiology. Principle among these is the stabilization of hypoxia-inducible factor- (HIF-) 1*α* in an oxygen dependent manner between 0.5 and 5% [25] which may be due to the release of reactive oxygen species by the mitochondria that prevents its degradation [26]. This raises the concentration of constitutively expressed HIF-1*α* which when combined with HIF-1*β* forms an active transcription factor promoting cell survival [27]. A variety of genes are affected by this cascade including chemokine receptors and those that promote anaerobic respiration, reduce aerobic respiration, and are involved in notch signaling [27]. HIF-1*α* mRNA expression was elevated in adipose-derived MSC after 48 hours of culture in the presence of 1% O_2_ [12]. In light of this, it is not surprising that we found that hypoxia alters the gene expression and secretion of paracrine factors, and HIF-*α* may be involved in the response.

This study demonstrated that reducing the serum concentration from 20% to 5% caused an increase in the mRNA expression for MMP-2 but a significant decrease in the expression for VEGF (normoxia only), MIP-1*α* (hypoxia only), and MIP-1*β* (hypoxia only) while MCP-1 was unaffected. Secretion of MMP-2 and secretion of MIP-1*β* were both significantly elevated by serum reduction while VEGF and MCP-1 were significantly reduced and MIP-1*α* was unaffected. We also observed that serum reduction caused a decline in cell proliferation. Alterations in proliferation, gene expression, and secretion after serum reduction are not surprising since the media contain less protein and fewer signaling growth factors. Other studies have demonstrated that serum reduction (to 2%) or deprivation causes an increased expression and secretion of VEGF by MSC [20, 22, 23]. It is unclear why lowering serum reduced expression and secretion of VEGF in our study while others found it to be elevated, but this could be attributed to our use of serum that contained stimulatory supplements (Mesencult; Stem Cell Technology) instead of FBS. However, we have previously demonstrated that this supplement does not contain VEGF, MIP-1*α*, MIP-1*β*, or MCP-1 [2] which was confirmed by the manufacturer, so that the changes observed are not simply due to a reduction in endogenous VEGF. Reports from other laboratories have shown that reducing serum from 20% to 2% in MSC cultures resulted in a significant decline in proliferation, an increase in the secretion of hepatocyte growth factor [22], and an increased expression of genes for placental growth factor, angiogenin-1, and bFGF [20]. Serum deprivation led to an increase in the gene expression and secretion of IGF-1, leptin, and angiogenin [23, 28] by MSC. Since coronary artery occlusion results in a serum-deprived state in the myocardium, these results have significant therapeutic implications for the paracrine actions of MSC.

Hypoxia and serum reduction also affect the genes associated with differentiation in MSC. Hypoxia increased the expression of the pluripotent genes OCT-4 and nanog in MSC [29] and stimulated the expression of a group of genes that maintained their undifferentiated state [30]. Hypoxia promoted osteogenic [31] and chondrogenic differentiation of MSC [32] but reduced adipogenic differentiation of MSC [15, 16] and promoted development of the intervertebral disc phenotype [14]. Reduction of serum levels to 2% caused an increased expression of “stemness” genes (ATP binding cassette G-2, bone marrow stromal cell antigen-1, FGF-4, frizzled-9, SOX-2, and OCT-4), angiogenic genes (placental growth factor, VEGF, angiogenin-1, and bFGF), and endogenic genes (CD34, vascular endothelial cadherin, endothelial cell adhesion molecule-1, von Wildebrand factor, and VEGF receptor-2) in MSC [20]. Serum reduction led to increased cardiomyocyte [19] and endothelial differentiation [21, 23] by MSC. Therefore, the hypoxic/low serum conditions present after coronary artery occlusion provide conditions that maintain the pluripotency of MSC while also promoting their ability to differentiate. This change in the differentiation state might also affect the expression and secretion of paracrine factors.

The paracrine factors secreted by MSC have significant biologic activities that are believed to play a role in their cardioprotective effects [3]. We have demonstrated that MSC conditioned media (CM) promote angiogenesis in canine vascular endothelial cells, MCP-1 or MSC-CM reduced caspase-3 activity in H9c2 cells, and MCP-1 and MIP-1*α* promoted MSC migration and VEGF inhibited MSC migration [2]. VEGF is a well-known promoter and regulator of angiogenesis [33], and MCP-1 has proangiogenic effects as well [34, 35]. MCP-1 [36], MIP-1*α*, and MIP-1*β* [37, 38] play a role in the modulation of immune function, and immune modulation may be an important mechanism whereby MSC aid tissues in recovery from injury [39] including myocardial infarction [40]. MMP-2 is a gelatinase that digests collagen and is tightly regulated by a variety of factors including the tissue inhibitors of metalloproteinases [41] which are also secreted by MSC [42]. The secretion of MMP-2 by MSC that we and others have demonstrated may be important in the transendothelial and tissue migration of MSC [24, 43] and in angiogenesis [44]. Differential regulation of these paracrine factors by hypoxic and low serum conditions as demonstrated in this study may be important in the beneficial effects of MSC injection during the ischemia present following coronary artery occlusion [2].

## Figures and Tables

**Table 1:** PCR primers.

	Forward	Reverse
VEGF	CAGGCTGCTGTAACGATGAA	TGTCTTTCTTTGGTCTGCATTC
MCP-1	AGGTCCCTGTCATGCTTCTG	TCTGGACCCATTCCTTCTTG
MIP-1*α*	TGCCCTTGCTGTTCTTCTCT	CCCAGGTCTCTTTGGAGTCA
MIP-1*β*	TGTCTGCCCTCTCTCTCCTC	GTCTGCCTCTTTTGGTCAGG
MMP-2	CAGGGCACCTCCTACAACAG	GTCAGTATCAGCATCGGGGG
YWHAZ	AACAGCTTTCGATGAAGCCAT	TGGGTATCCGATGTCCACAAT

**Table 2:** Paracrine factors in media.

	Normoxia		Hypoxia	
	5%	20%	5%	20%
VEGF (*n* = 6)	1447.7 ± 93.5^a^	2102.37 ± 138.7	1601.17 ± 66.3^a^	2247.37 ± 83.6
MCP-1 (*n* = 6)	3964.4 ± 244.9^a^	4985.1 ± 160.1	1880.0 ± 141.3^ab^	2705.7 ± 143.8^b^
MIP-1*α* (*n* = 6)	5.8 ± 0.4	6.2 ± 0.2	5.3 ± 0.5	6.0 ± 0.4
MIP-1*β* (*n* = 5)	5.1 ± 0.6	4.1 ± 0.1	4.0 ± 0.1^ab^	3.5 ± 0.2^b^
MMP-2 (*n* = 6)	37.3 ± 1.0^a^	26.8 ± 2.2	28.6 ± 1.3^ab^	19.7 ± 0.2^b^

Data reported as mean ± SE in pg/mL except for MMP-2 (ng/mL). 5% and 20% refer to the amount of serum supplement present in the media. ^a^ *P* < 0.05 versus 20%; ^b^ *P* < 0.05 versus normoxia.

**Table 3:** Normalized relative expression of mRNA for paracrine factors.

	Normoxia		Hypoxia	
	5%	20%	5%	20%
VEGF (*n* = 5)	0.535 ± 0.038^a^	0.667 ± 0.067	0.725 ± 0.051^b^	0.812 ± 0.085
MCP-1 (*n* = 5)	1.162 ± 0.145	1.410 ± 0.231	0.259 ± 0.063^b^	0.557 ± 0.214^b^
MIP-1*α* (*n* = 5)	0.688 ± 0.042	0.757 ± 0.051	1.196 ± 0.136^ab^	1.578 ± 0.061^b^
MIP-1*β* (*n* = 5)	0.690 ± 0.069	0.600 ± 0.036	0.932 ± 0.101^ab^	1.290 ± 0.127^b^
MMP-2 (*n* = 11)	0.855 ± 0.039^a^	0.583 ± 0.064	0.886 ± 0.066	0.718 ± 0.135

Data reported as mean ± SE. 5% and 20% refer to the amount of serum supplement present in the media. ^a^ *P* < 0.05 versus 20%; ^b^ *P* < 0.05 versus normoxia.

## References

[1] Boomsma R. A., Swaminathan P. D., Geenen D. L. (2007). Intravenously injected mesenchymal stem cells home to viable myocardium after coronary occlusion and preserve systolic function without altering infarct size. *International Journal of Cardiology*.

[2] Boomsma R. A., Geenen D. L. (2012). Mesenchymal stem cells secrete multiple cytokines that promote angiogenesis and have contrasting effects on chemotaxis and apoptosis. *PLoS ONE*.

[3] Burchfield J. S., Dimmeler S. (2008). Role of paracrine factors in stem and progenitor cell mediated cardiac repair and tissue fibrosis. *Fibrogenesis and Tissue Repair*.

[4] Tsai C. C., Yew T. L., Yang D. C., Huang W. H., Hung S. C. (2012). Benefits of hypoxic culture on bone marrow multipotent stromal cells. *American Journal of Blood Research*.

[5] Hoffman W. E., Albrecht R. F., Ripper R., Jonjev Z. S. (2001). Brain compared to heart tissue oxygen pressure during changes in arterial carbon dioxide in the dog. *Journal of Neurosurgical Anesthesiology*.

[6] Campbell J. A. (1925). The influence of O_2_-tension in the inspired air upon the O_2_-tension in the tissues. *The Journal of Physiology*.

[7] Caldwell C. C., Kojima H., Lukashev D. (2001). Differential effects of physiologically relevant hypoxic conditions on T lymphocyte development and effector functions. *The Journal of Immunology*.

[8] Khan M., Kutala V. K., Vikram D. S. (2007). Skeletal myoblasts transplanted in the ischemic myocardium enhance in situ oxygenation and recovery of contractile function. *The American Journal of Physiology—Heart and Circulatory Physiology*.

[9] Ivanovic Z. (2009). Hypoxia or in situ normoxia: the stem cell paradigm. *Journal of Cellular Physiology*.

[10] Ohnishi S., Yasuda T., Kitamura S., Nagaya N. (2007). Effect of hypoxia on gene expression of bone marrow-derived mesenchymal stem cells and mononuclear cells. *Stem Cells*.

[11] Hung S.-C., Pochampally R. R., Chen S.-C., Hsu S.-C., Prockop D. J. (2007). Angiogenic effects of human multipotent stromal cell conditioned medium activate the PI3K-Akt pathway in hypoxic endothelial cells to inhibit apoptosis, increase survival, and stimulate angiogenesis. *Stem Cells*.

[12] Liu L., Gao J., Yuan Y., Chang Q., Liao Y., Lu F. (2013). Hypoxia preconditioned human adipose derived mesenchymal stem cells enhance angiogenic potential via secretion of increased VEGF and bFGF. *Cell Biology International*.

[13] Burlacu A., Grigorescu G., Rosca A.-M., Preda M. B., Simionescu M. (2013). Factors secreted by mesenchymal stem cells and endothelial progenitor cells have complementary effects on angiogenesis *in vitro*. *Stem Cells and Development*.

[14] Stoyanov J. V., Gantenbein-Ritter B., Bertolo A. (2011). Role of hypoxia and growth and differentiation factor-5 on differentiation of human mesenchymal stem cells towards intervertebral nucleus pulposus-like cells. *European Cells & Materials*.

[15] Hung S.-P., Ho J. H., Shih Y.-R. V., Lo T., Lee O. K. (2012). Hypoxia promotes proliferation and osteogenic differentiation potentials of human mesenchymal stem cells. *Journal of Orthopaedic Research*.

[16] Wagegg M., Gaber T., Lohanatha F. L. (2012). Hypoxia promotes osteogenesis but suppresses adipogenesis of human mesenchymal stromal cells in a hypoxia-inducible factor-1 dependent manner. *PLoS ONE*.

[17] Bialik S., Cryns V. L., Drincic A. (1999). The mitochondrial apoptotic pathway is activated by serum and glucose deprivation in cardiac myocytes. *Circulation Research*.

[18] Bonavita F., Stefanelli C., Giordano E. (2003). H9c2 cardiac myoblasts undergo apoptosis in a model of ischemia consisting of serum deprivation and hypoxia: inhibition by PMA. *FEBS Letters*.

[19] Grajales L., García J., Banach K., Geenen D. L. (2010). Delayed enrichment of mesenchymal cells promotes cardiac lineage and calcium transient development. *Journal of Molecular and Cellular Cardiology*.

[20] Chua K. H., Raduan F., Safwani W. K. Z. W., Manzor N. F. M., Pingguan-Murphy B., Sathapan S. (2013). Effects of serum reduction and VEGF supplementation on angiogenic potential of human adipose stromal cells in vitro. *Cell Proliferation*.

[21] Follin B., Tratwal J., Haack-Sørensen M., Elberg J. J., Kastrup J., Ekblond A. (2013). Identical effects of VEGF and serum-deprivation on phenotype and function of adipose-derived stromal cells from healthy donors and patients with ischemic heart disease. *Journal of Translational Medicine*.

[22] Katsuno T., Ozaki T., Saka Y. (2013). Low serum cultured adipose tissue-derived stromal cells ameliorate acute kidney injury in rats. *Cell Transplantation*.

[23] Oskowitz A., McFerrin H., Gutschow M., Carter M. L., Pochampally R. (2011). Serum-deprived human multipotent mesenchymal stromal cells (MSCs) are highly angiogenic. *Stem Cell Research*.

[24] Ries C., Egea V., Karow M., Kolb H., Jochum M., Neth P. (2007). MMP-2, MT1-MMP, and TIMP-2 are essential for the invasive capacity of human mesenchymal stem cells: differential regulation by inflammatory cytokines. *Blood*.

[25] Jiang B.-H., Semenza G. L., Bauer C., Marti H. H. (1996). Hypoxia-inducible factor 1 levels vary exponentially over a physiologically relevant range of O_2_ tension. *American Journal of Physiology*.

[26] Guzy R. D., Schumacker P. T. (2006). Oxygen sensing by mitochondria at complex III: the paradox of increased reactive oxygen species during hypoxia. *Experimental Physiology*.

[27] Haque N., Rahman M. T., Abu Kasim N. H., Alabsi A. M. (2013). Hypoxic culture conditions as a solution for mesenchymal stem cell based regenerative therapy. *The Scientific World Journal*.

[28] Sanchez C., Oskowitz A., Pochampally R. R. (2009). Epigenetic reprogramming of IGF1 and leptin genes by serum deprivation in multipotential mesenchymal stromal cells. *Stem Cells*.

[29] Tsai C.-C., Su P.-F., Huang Y.-F., Yew T.-L., Hung S.-C. (2012). Oct4 and Nanog directly regulate Dnmt1 to maintain self-renewal and undifferentiated state in mesenchymal stem cells. *Molecular Cell*.

[30] Basciano L., Nemos C., Foliguet B. (2011). Long term culture of mesenchymal stem cells in hypoxia promotes a genetic program maintaining their undifferentiated and multipotent status. *BMC Cell Biology*.

[31] Lennon D. P., Edmison J. M., Caplan A. I. (2001). Cultivation of rat marrow-derived mesenchymal stem cells in reduced oxygen tension: effects on *in vitro* and *in vivo* osteochondrogenesis. *Journal of Cellular Physiology*.

[32] Martin-Rendon E., Hale S. J. M., Ryan D. (2007). Transcriptional profiling of human cord blood CD133+ and cultured bone marrow mesenchymal stem cells in response to hypoxia. *Stem Cells*.

[33] Shibuya M. (2013). Vascular endothelial growth factor and its receptor system: physiological functions in angiogenesis and pathological roles in various diseases. *Journal of Biochemistry*.

[34] Ma J., Wang Q., Fei T., Han J.-D. J., Chen Y.-G. (2007). MCP-1 mediates TGF-*β*-induced angiogenesis by stimulating vascular smooth muscle cell migration. *Blood*.

[35] Salcedo R., Ponce M. L., Young H. A. (2000). Human endothelial cells express CCR2 and respond to MCP-1: direct role of MCP-1 in angiogenesis and tumor progression. *Blood*.

[36] Deshmane S. L., Kremlev S., Amini S., Sawaya B. E. (2009). Monocyte chemoattractant protein-1 (MCP-1): an overview. *Journal of Interferon and Cytokine Research*.

[37] Maurer M., von Stebut E. (2004). Macrophage inflammatory protein-1. *The International Journal of Biochemistry & Cell Biology*.

[38] Menten P., Wuyts A., Van Damme J. (2002). Macrophage inflammatory protein-1. *Cytokine and Growth Factor Reviews*.

[39] English K. (2013). Mechanisms of mesenchymal stromal cell immunomodulation. *Immunology and Cell Biology*.

[40] Van Den Akker F., Deddens J. C., Doevendans P. A., Sluijter J. P. G. (2013). Cardiac stem cell therapy to modulate inflammation upon myocardial infarction. *Biochimica et Biophysica Acta: General Subjects*.

[41] Visse R., Nagase H. (2003). Matrix metalloproteinases and tissue inhibitors of metalloproteinases: structure, function, and biochemistry. *Circulation Research*.

[42] Lozito T. P., Jackson W. M., Nesti L. J., Tuan R. S. (2014). Human mesenchymal stem cells generate a distinct pericellular zone of MMP activities via binding of MMPs and secretion of high levels of TIMPs. *Matrix Biology*.

[43] De Becker A., Van Hummelen P., Bakkus M. (2007). Migration of culture-expanded human mesenchymal stem cells through bone marrow endothelium is regulated by matrix metalloproteinase-2 and tissue inhibitor of metalloproteinase-3. *Haematologica*.

[44] Stetler-Stevenson W. G., Seo D.-W. (2005). TIMP-2: an endogenous inhibitor of angiogenesis. *Trends in Molecular Medicine*.

